# Supplementary figures and images for: Discrimination of biofilm-producing Stenotrophomonas maltophilia clinical strains by matrix-assisted laser desorption ionization–time of flight
Source: PLoS One. 2020 Dec 31;15(12):e0244751. doi: 10.1371/journal.pone.0244751 (PMC7775041; doi:10.1371/journal.pone.0244751)

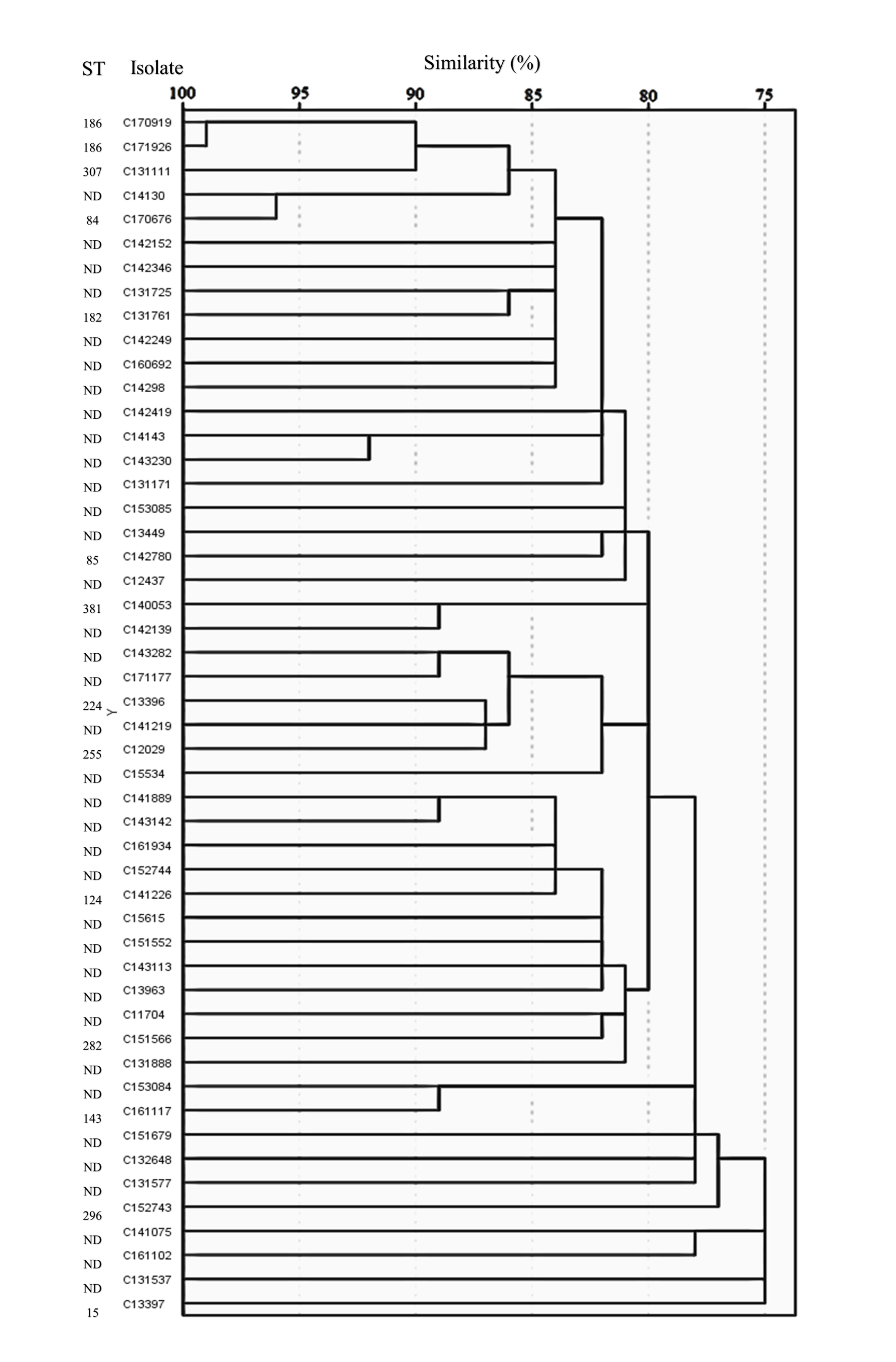

Supplement: S1 Fig — Fifty S. maltophilia isolates obtained from the ICU from both hospitals were analyzed by PFGE and MLST. ICU: Intensive care unit; ND: Not determined. (TIF) [file pone.0244751.s001.tif]
