# Supplementary material for: Discrimination of biofilm-producing Stenotrophomonas maltophilia clinical strains by matrix-assisted laser desorption ionization–time of flight
Source: PLoS One. 2020 Dec 31;15(12):e0244751. doi: 10.1371/journal.pone.0244751 (PMC7775041; doi:10.1371/journal.pone.0244751)
Supplement: Attachment — Submitted filename: Response to Reviewers.docx [file pone.0244751.s003.docx]

Joseph Banoub, PhD., D. Sc.

Academic Editor

PLOS ONE

Dear Editor:

Please find the corrections and responses to the comments kindly given to the manuscript entitled “Discrimination of biofilm-producing *Stenotrophomonas maltophilia* clinical strains by matrix-assisted laser desorption ionization–time of flight” that we submitted to the *PLOS ONE* journal for consideration as a full-length paper (Reference number PONE-D-20-31198).

We thank the Editor and the Reviewer for the time and suggested corrections. We think that these modifications significantly improve the manuscript and we hope they are to the both Editor and Reviewer liking.

Sincerely,

Samantha Flores-Treviño (samflorest@gmail.com)

Universidad Autónoma de Nuevo León

**Editor Comments to the Author**

Include the complete MALDI-TOF-MS and indicate with different colors your three biomarkers at *m/z* 4661, 6074 and 6102.

**Response:** We included a new figure with three complete mass spectra from three isolates with different genotype profiles. Fig 1. Representative mass spectra comparison of potential biomarker peaks of *rmlA^+^* and *rpF-2^−^* genotype in biofilm-producing *S. maltophilia* isolates.

1. Please ensure that your manuscript meets PLOS ONE's style requirements, including those for file naming.

**Response:** We have changed file names accordingly to meet PLOS ONE's style requirements.

2. Please note that PLOS does not permit references to “data not shown.” Authors should provide the relevant data within the manuscript, the Supporting Information files, or in a public repository. If the data are not a core part of the research study being presented, we ask that authors remove any references to these data.

**Response:** We have made the changes accordingly in lines 258-259 and lines 314-315.

3. In your Methods section, please provide additional information about the medical data/samples collected and the demographic details of the human subjects. Please ensure you have provided sufficient details to replicated the analyses such as: a) a description of any inclusion/exclusion criteria that were applied to the medical records/samples, b) a table of relevant demographic details.

**Response:**  The following supplementary table was included: S1 Table. Clinical and demographic characteristics of patients included in the study. The results obtained regarding biofilm production, EPS major composition, presence of biofilm-associated genes, and potential biofilm biomarker peaks are also included.

4. To comply with PLOS ONE submission guidelines, in your Methods section, please provide additional information regarding your statistical analyses.

**Response:** We have expanded the Methods section including Lines 184─188, and Lines 196─209.

**Reviewer #1 Comments to the Author**

1. Line 161 and Line 356, UCI-obtained? A typo?

**Response:** Yes, it is indeed a typo. We apologize for the inconvenience. We have corrected the typo from UCI to ICU.

2. Line 176, Please include a reference for BLAST program.

**Response:** We have added the following reference: Altschul SF, Gish W, Miller W, Myers EW, Lipman DJ. Basic local alignment search tool. J Mol Biol. 1990;215(3):403-10. doi: 10.1016/S0022-2836(05)80360-2. PubMed PMID: 2231712.

3. Line 214, the supplementary Figure is not provided.

**Response:** We apologize for the inconvenience. The supplementary Figure S1 Fig has been added to the Supplementary files.

4. In Table 1, please state why does the number in the parentheses stand for?

**Response:** The number inside the parenthesis stands for the percentage out of the total analyzed strains, which is 120. This total number is included in the text and in the Table title.

5. In Table 1, please use rpfF-2 for consistency.

**Response:** We have made corrected rpf to rpf-2.

6. In Table 1, does the term "Other" mean that an unknown component other than polysaccharides, proteins, and DNA play a major role in the biofilm?

**Response:** Yes, “Other” refers to EPS components different from polysaccharides, proteins, and DNA. We have changed the term to “Unknown” to avoid confusion.
